# Pre-diagnostic clinical features and blood tests in patients with colorectal cancer: a retrospective linked-data study

**DOI:** 10.3399/BJGP.2021.0563

**Published:** 2022-06-07

**Authors:** Marie Moullet, Garth Funston, Luke TA Mounce, Gary A Abel, Niek de Wit, Fiona M Walter, Yin Zhou

**Affiliations:** Primary Care Unit, Department of Public Health and Primary Care, University of Cambridge, Cambridge, UK.; Primary Care Unit, Department of Public Health and Primary Care, University of Cambridge, Cambridge; Centre for Primary Care and Health Service Research, University of Manchester, Manchester, UK.; University of Exeter Medical School, University of Exeter, Exeter, UK.; University of Exeter Medical School, University of Exeter, Exeter, UK.; Julius Center for Health Science and Primary Care, University Medical Centre Utrecht, Utrecht University, the Netherlands.; Primary Care Unit, Department of Public Health and Primary Care, University of Cambridge, Cambridge; Wolfson Institute of Population Health, Queen Mary University of London, London, UK.; Primary Care Unit, Department of Public Health and Primary Care, University of Cambridge, Cambridge, UK.

**Keywords:** colon cancer, early diagnosis, primary health care, rectal cancer, retrospective studies, general practice

## Abstract

**Background:**

The majority of colorectal cancer is diagnosed in patients following symptomatic presentation in the UK.

**Aim:**

To identify windows of opportunity for timely investigations or referrals in patients presenting with colon and rectal cancer-relevant symptoms or abnormal blood tests.

**Design and setting:**

A retrospective cohort study was undertaken using linked primary care and cancer registry data for patients with colorectal cancer diagnosed in England between 2012 and 2015.

**Method:**

Monthly consultation rates for relevant clinical features (change in bowel habit, rectal bleeding, abdominal pain, abdominal mass, constitutional symptoms, and other bowel symptoms) and abnormal blood test results (low haemoglobin, high platelets, and high inflammatory markers) up to 24 months pre-diagnosis were calculated. Poisson regression adjusted for age, sex, and relevant comorbidities was used to estimate the most likely month when consultation rates increased above baseline.

**Results:**

In total, 5033 patients with colon cancer and 2516 with rectal cancer were included. Consultations for all examined clinical features and abnormal blood tests increased in the year pre-diagnosis. Rectal bleeding was the earliest clinical feature to increase from the baseline rate: at 10 months (95% confidence interval [CI] = 8.3 to 11.7) pre-diagnosis for colon cancer and at 8 months (95% CI = 6.1 to 9.9) pre-diagnosis for rectal cancer. Low haemoglobin, high platelets, and high inflammatory markers increased from as early as 9 months pre-diagnosis.

**Conclusion:**

This study found evidence for an early increase in rates of consultation for relevant clinical features and abnormal blood tests in patients with colorectal cancer, suggesting that earlier instigation of cancer-specific investigations or referrals may be warranted in some patients who were symptomatic.

## INTRODUCTION

Colorectal cancer is the fourth most common cancer and the second most common cause of cancer-related deaths in the UK.^1^ Despite the existence of bowel cancer screening programmes, the majority (about 53%) of cases of colorectal cancer are diagnosed in patients following symptomatic presentation in primary care.^2^ Timely diagnosis following symptomatic presentation matters because earlier detection allows earlier treatment and improved outcomes, with better survival when cancer is diagnosed at an earlier stage.^3^

In the UK, most patients with undiagnosed colorectal cancer first present to GPs.^2^ Patients with alarm symptoms of colorectal cancer, including rectal bleeding, change in bowel habit, rectal or abdominal mass, and unexplained anaemia, can be fast-tracked for assessment by a specialist within 14 days under the 2-week-wait system (hereafter known as fast-track referral), based on National Institute for Health and Care Excellence (NICE) guidelines.^4^^,^^5^ However, not all patients experience alarm symptoms in the year leading up to colorectal cancer diagnosis. Many patients with colorectal cancer report non-specific gastrointestinal and constitutional symptoms such as abdominal pain, weight loss, or fatigue in the years before diagnosis.^6^^,^^7^ They also have higher consultation rates for musculoskeletal, neurological, respiratory, and endocrine dysfunction than matched controls.^8^ The low positive predictive values of these less specific symptoms pose a challenge for timely diagnosis. The faecal immunochemical test (FIT), which is now available in UK general practice,^9^ may be a useful test to triage lower-risk patients with possible colorectal cancer for further investigations or referral.

Existing evidence demonstrates that clinical activities such as consultation rates increase before cancer diagnosis, suggesting that opportunities may exist to initiate investigations sooner, and therefore expedite diagnosis, in some patients with cancer.^10^^–^^12^ In Denmark, patients with colorectal cancer had higher overall consultation rates than matched controls as early as 9 months before diagnosis.^13^ Prescriptions for any medication and specifically for haemorrhoid medications,^8^ and performance of haemoglobin tests,^13^ were also higher in patients with colorectal cancer than matched controls in the year leading up to diagnosis.

**Table:** How this fits in

Understanding pre-diagnostic patterns of relevant clinical features and abnormal blood test results in patients with colon and rectal cancer could elucidate windows of opportunity during which more timely investigations and referrals could be performed, and earlier diagnosis of cancer could be achieved. This study found that consultation rates increased in the year leading up to diagnosis for relevant clinical features such as low haemoglobin, rectal bleeding, and change in bowel habits, as well as non-specific blood tests, from as early as 9–10 months before diagnosis. These findings suggest that potential opportunities for more timely use of cancer investigations or referral exist, and could improve diagnostic pathways, expediting diagnosis and treatment for some patients with colorectal cancer.

In addition to clinical activities, cohort studies in UK primary care have identified non-specific blood-based biomarkers associated with increased risk of cancer in general, including high platelet counts^14^ and markers of inflammation.^15^ It is not currently known if and with what frequency these generic abnormal blood tests occur in the pre-diagnostic period in patients with colorectal cancer. Examining pre-diagnostic patterns of these abnormal test results may be helpful for informing clinicians of the possible time during which these generic tests first become abnormal, which may represent the first signals of possible colorectal cancer, therefore prompting clinicians to arrange for suitable and timely follow-up or investigations as appropriate.

Against this background, this study aims to provide a comprehensive and up-to-date description of the pattern of relevant symptoms and abnormal blood tests in patients with colorectal cancer in the months leading up to diagnosis, and to identify when first signals of possible colorectal cancer might occur, so that timely investigations can be initiated.

## METHOD

Linked primary care data from the Clinical Practice Research Datalink (CPRD) GOLD and National Cancer Registration Analysis Service (NCRAS) that included all patients with a first record of colorectal cancer recorded in CPRD between 1 April 2012 and 31 December 2015 was used. The cohort was supplemented with all patients with colorectal cancer recorded in the Cancer Registry using *International Classification of Diseases, 10th Revision* (ICD-10) codes C18 (colon cancer) and C19–C20 (rectal cancer; colorectal cancer diagnosis codes are reported in Supplementary Table S1). When the diagnosis or date differed between the CPRD and the Cancer Registry, Cancer Registry data were retained.

Colorectal cancer symptom and gastrointestinal comorbidity code lists were derived from previously published studies.^7^^,^^16^^–^^18^ Lists were cross-checked by two of the authors (English GPs) and categorised into clinically relevant groups for analysis. Relevant symptom categories, including alarm symptoms drawn from the NICE 2015 cancer referral guidelines, were included.^5^ After discussion with clinical co-authors, rectal bleeding; change in bowel habit (including constipation and diarrhoea); abdominal pain; constitutional symptoms (including fatigue, appetite loss, and weight loss, combined because of the low frequency of individual symptoms); and other bowel symptoms (including bloating, flatulence, wind, and obstruction) were included (see Supplementary Table S2 for code lists). The choice of constitutional symptoms and other bowel symptoms were chosen because of their likelihood of triggering clinical and cancer-excluding investigations (including FIT), based on the experience of the clinical co-authors.

Relevant comorbidities were selected and grouped based on previous literature^19^ and clinical consensus among the co-authors. Group 1 included inflammatory bowel disease (IBD) and diverticular disease, which are associated with increased risk of colorectal cancer.^20^ Group 2 included irritable bowel disease (IBS), coeliac, and gall bladder disease, which may mimic colorectal cancer presentations and present diagnostic challenges. Patients were counted as having IBD, diverticular, coeliac, or gall bladder disease if they ever had a recording for one of these diseases, and IBS if they had a diagnosis prior to 2 years before cancer diagnosis (because of possible misdiagnosis of IBS in the 2 years immediately before colorectal cancer diagnosis^17^). Group 3 included patients with haemorrhoids in the 5 years leading up to diagnosis, as these patients were likely to have had rectal bleeding but not be referred on a fast-track pathway owing to them being given an alternative non-malignant diagnosis. Code lists for all comorbidities are presented in Supplementary Table S3.

Based on previously reported associations with cancer, blood test results for platelets, C-reactive protein (CRP), erythrocyte sedimentation rate (ESR), and haemoglobin were analysed.^15^^,^^16^^,^^21^ A decision was taken to focus on the key blood tests in the literature, and for which it was possible to make more reliable inferences because of their larger sample sizes. Cut-offs for normal values were taken from NICE guidelines^5^ or the literature.^14^^,^^15^ Patients were regarded as having raised inflammatory markers if either CRP or ESR were abnormal. Reference ranges for each marker are described in Box 1.

**Box 1.** Blood markers included in this study and thresholds

**Marker**	**Threshold (source)**
Haemoglobin	<110 g/L for males, <100 g/L for females^5^
Platelets	>400 × 10^9^/L^5^
C-reactive protein	>7 mg/L^15^
Erythrocyte sedimentation rate	Previously defined age-and sex-specific thresholds^15^

The rates of recordings for each clinical feature and abnormal blood test up to 2 years before diagnosis were described first, as a previous study comparing clinical activity in patients with colorectal cancer and controls identified differences emerging as early as 17 months pre-diagnosis.^13^ A series of 24 multilevel Poisson regression models were constructed to identify the most likely month (28-day period) when cohort-level rates of clinical-feature recordings increased above baseline. Each model included a continuous month term, to account for any background trend, and a second ‘inflection month’ variable to capture deviation from the background trend. This second variable was equal to the number of months between the specified inflection point for that model and the month of interest for months between the inflection point and diagnosis, and equal to zero otherwise. The month with the model corresponding to the largest log likelihood was selected and considered the best-fitting model, with confidence intervals (CIs) for this month provided via bootstrapping. Adjustments were made in all models for age, sex, relevant comorbidity groups, and month pre-diagnosis. Colon and rectal cancer were considered separately in the analysis because they can present differently.^7^

All analysis was carried out in Stata/IC (version 16.1), graphs were drawn using R (version 3.6.1) and the *ggplot* package (version 3.3.5).

## RESULTS

In total, 7549 patients were included in the study, consisting of 5033 (66.7%) with colon and 2516 (33.3%) with rectal cancer, respectively. The proportions of baseline demographic characteristics by cancer site are shown in Table 1.

**Table 1.** Population demographic characteristics, by diagnosis

	**Cancer diagnosis**				**Total**	

	**Colon**		**Rectal**			

**Characteristic**	***n***	**%**	***n***	**%**	***n***	**%**
**Total**	5033	100	2516	100	7549	100

**Sex**						
Male	2624	52.1	1561	62.0	4185	55.4
Female	2409	47.9	955	38.0	3364	44.6

**Age group, years**						
25–59	849	16.9	578	23.0	1427	18.9
60–69	1200	23.8	721	28.7	1921	25.4
70–79	1533	30.5	729	29.0	2262	30.0
≥80	1451	28.8	488	19.4	1939	25.7

**Relevant comorbidities**						
Group 1: inflammatory bowel disease and diverticular disease	926	18.4	341	13.6	1267	16.8
Group 2: irritable bowel syndrome, coeliac, and gall bladder disease	316	6.3	102	4.1	418	5.5
Group 3: haemorrhoids	615	12.2	226	9.0	841	11.1

In the year pre-diagnosis, the most frequently recorded symptom was abdominal pain (25.9%, *n* = 1305/5033) in patients with colon cancer and it was rectal bleeding (32.2%, *n* = 810/2516) in the patients with rectal cancer (Table 2). The most frequent abnormal blood test result in the year pre-diagnosis was low haemoglobin in patients with colon cancer (28.2%, *n* = 1417/5033) and high inflammatory markers in patients with rectal cancer (18.4%, *n* = 463/2516).

**Table 2.** Prevalence of clinical-feature recordings in each pre-diagnostic period

	**Cancer diagnosis**				**Total (*N* = 7549)**	

	**Colon** **(*n* = 5033)**		**Rectal (*n* = 2516)**			

**Variable**	***n***	**%**	***n***	**%**	***n***	**%**
**Clinical features**						
**Abdominal pain**						
0–1 year	1305	25.9	271	10.8	1576	20.9
1–2 years	262	5.2	72	2.9	334	4.4
**Change in bowel habit**						
0–1 year	1091	21.7	787	31.3	1878	24.9
1–2 years	236	4.7	117	4.7	353	4.7
**Rectal bleeding**						
0–1 year	584	11.6	810	32.2	1394	18.5
1–2 years	82	1.6	63	2.5	145	1.9
**Constitutional symptoms**						
0–1 year	436	8.7	123	4.9	559	7.4
1–2 years	170	3.4	47	1.9	217	2.9
**Other bowel function**						
0–1 year	165	3.3	57	2.3	222	2.9
1–2 years	41	0.8	17	0.7	58	0.8
**Abdominal mass**						
0–1 year	83	1.6	21	0.8	104	1.4
1–2 years	7	0.1	5	0.2	12	0.2

**Abnormal blood tests**						
**Low haemoglobin**						
0–1 year	1417	28.2	231	9.2	1648	21.8
1–2 years	213	4.2	55	2.2	268	3.6
**High inflammatory markers**						
0–1 year	1392	27.7	463	18.4	1855	24.6
1–2 years	367	7.3	127	5.0	494	6.5
**High platelets**						
0–1 year	932	18.5	244	9.7	1176	15.6
1–2 years	142	2.8	52	2.1	194	2.6

a *Abdominal mass includes codes for abdominal and rectal or perianal masses.*

**Figure 1.**
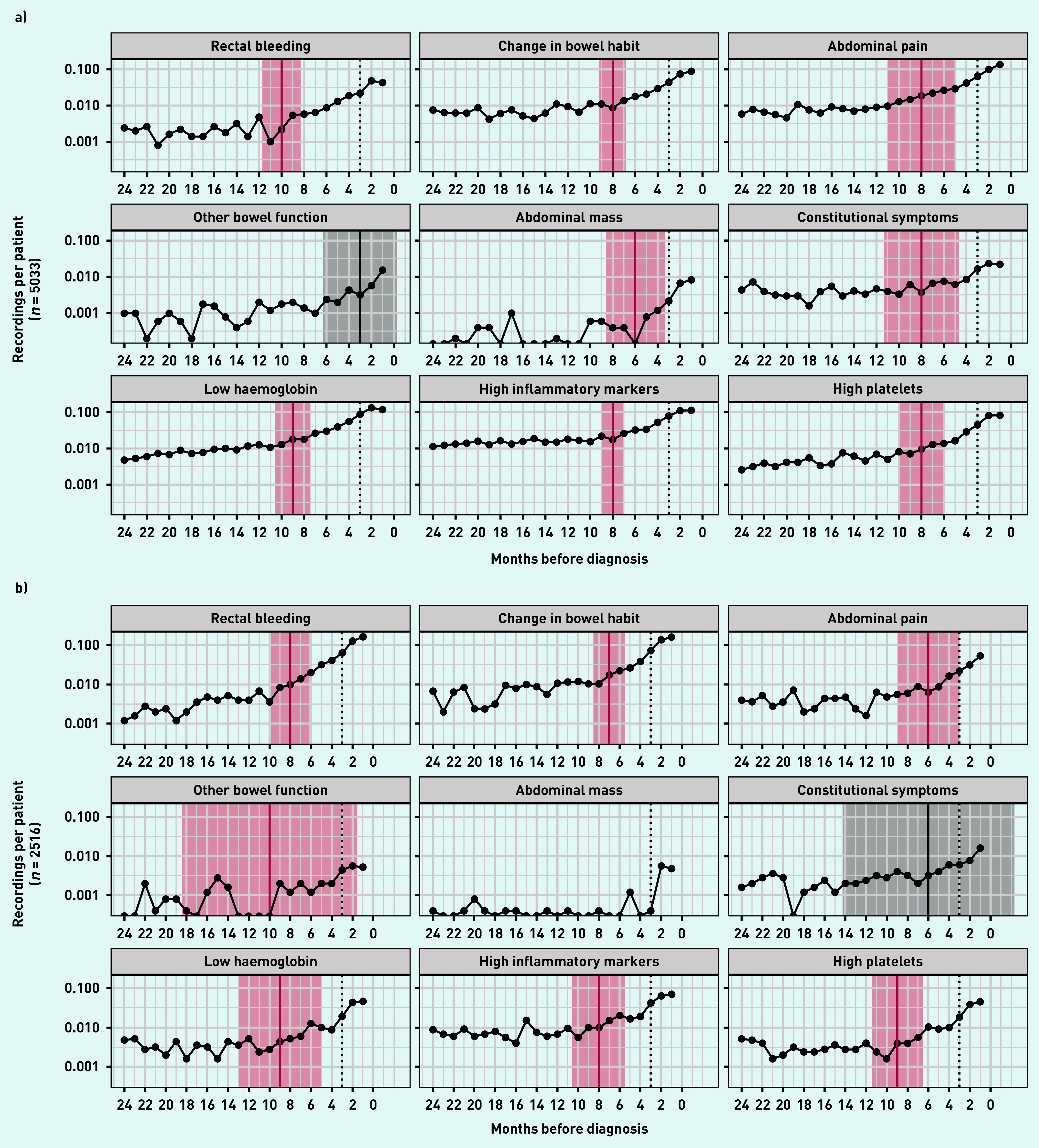
*Rates of recordings of each clinical feature in the 2 years leading up to diagnosis, by cancer diagnosis: a) colon cancer; b) rectal cancer. A solid vertical line indicates the most likely inflection point, shaded areas represents 95% confidence interval (red when confidence interval excludes 0, grey otherwise). Dotted vertical line represents 3 months before diagnosis. Inflection points are estimated in models adjusted for age, sex, and comorbidities. Months represent 28-day periods.*

In both colon and rectal cancer there was an increasing rate of recordings for rectal bleeding, change in bowel habit, abdominal pain, other bowel function, abdominal mass, and constitutional symptoms in the year before diagnosis. Similarly, the rate of recorded abnormal blood tests that were examined (low haemoglobin, high inflammatory markers, and high platelets) increased towards diagnosis during the same period (Figure 1).

In patients with colon cancer, rectal bleeding was the earliest clinical feature to increase from baseline, at 10 months before diagnosis (95% CI = 8.3 to 11.7) (Figure 1). This was followed by change in bowel habit (at 8 months; 95% CI = 6.8 to 9.2), abdominal pain (at 8 months; 95% CI = 5.0 to 11.0), and constitutional symptoms (at 8 months; 95% CI = 4.6 to 11.4), then abdominal mass (6 months; 95% CI = 3.4 to 8.6) and other bowel function (3 months; 95% CI = −0.3 to 6.3). Among the blood tests, rate of low haemoglobin increased from 9 months pre-diagnosis (95% CI = 7.4 to 10.6). The rate of high platelets and high inflammatory markers increased from 8 months (high platelets 95% CI = 6.0 to 10.0, high inflammatory markers 95% CI = 7.1 to 8.9) pre-diagnosis.

In patients with rectal cancer, the earliest inflection point estimate was for other bowel function, albeit this estimate had a large CI (10 months; 95% CI = 1.5 to 18.5). This was followed by rectal bleeding (8 months; 95% CI = 6.1 to 9.9), change in bowel habit, and abdominal pain (7 months; 95% CI = 5.5 to 8.5, and 6 months; 95% CI = 3.0 to 9.0 pre-diagnosis, respectively). Among blood tests, low haemoglobin and high platelets increased from baseline as early as 9 months pre-diagnosis (low haemoglobin 95% CI = 5.0 to 13.0, high platelets 95% CI = 6.5 to 11.5). There was not statistical evidence for an increase above baseline for constitutional symptoms (estimated inflection point 6 months pre-diagnosis; 95% CI = −2.3 to 14.3). The number of observations for abdominal mass was too low to calculate an inflection point in patients with rectal cancer.

## DISCUSSION

### Summary

This study found increasing rates of consultation for gastrointestinal alarm symptoms and abnormal test results among patients diagnosed with colorectal cancer in the year before diagnosis. Rates of rectal bleeding increased as early as 10 months pre-diagnosis in patients with colon cancer, and 8 months pre-diagnosis in patients with rectal cancer. Low haemoglobin and changes in non-specific blood tests including high inflammatory markers and platelets were found to increase as early as 9 months pre-diagnosis in patients with colon and rectal cancer. These findings indicate that there may be opportunities to initiate specific cancer investigations or referrals sooner, and therefore expedite diagnosis and treatment in some patients with colorectal cancer.

### Strengths and limitations

The strength of this study includes using a large representative sample,^22^ and prospectively recorded electronic health records, which are not subject to recall or survivorship biases. Furthermore, results for the blood tests are automatically coded within CPRD when received from the laboratories, and are less likely to be subject to manual coding issues.

Despite including only patients who had cancer, the Poisson modelling allowed estimation of inflection points without data from non-cancer controls, and bootstrapping allowed estimation of 95% CIs, which are more informative than point estimates alone. Although it was not possible to determine the clinical indications for the performance of these blood tests and whether the patients with alarm symptoms met fast-track referral guidelines the analysis method used, which included adjustment for relevant comorbidities, made it possible to account for background rates of abnormal blood tests because of chronic disease monitoring or other existing conditions. Therefore, the authors believe the inflection points reflect new changes in rates of abnormal tests that may require further investigation or monitoring.

Coded data do not contain information about symptom severity or duration,^23^ and might be subject to clinician recording bias. A previous study using CPRD found that a third of all abdominal pain records were present as free text for patients with pancreatic or bladder cancer.^24^ Although these factors may contribute to underestimation of symptom prevalence, this study focused on changes in rates of recordings over time, and therefore the results are less affected by the effect of underestimation.

No statistically significant effect of deprivation (based on individual-level Index of Multiple Deprivation) on the rate of pre-diagnostic clinical features was found (results not shown). The effect of ethnicity was not examined because of the very small number of patients with ethnicity other than White recorded in this sample. Although not biasing the results of this study, this may limit the generalisability of the findings to other populations with a different ethnicity mix. Finally, it is important to note that these results only represent population-level signals of change in clinical activity, and do not relate to associations seen at an individual level, including the predictive values of the clinical features.

### Comparison with existing literature

Similar to previous studies looking at patterns of pre-diagnostic activities in patients with cancer in Denmark,^13^^,^^25^ the current study found increasing rates of consultation for relevant symptoms and abnormal tests in patients with colorectal cancer in the year before diagnosis. These previous studies had examined the overall number of consultations during the pre-diagnostic period.^13^^,^^25^ The current study enhanced existing evidence by examining consultation rates for relevant clinical symptoms, signs, and blood test results of patients with colorectal cancer. These findings therefore improve the characterisation of symptomatic presentations of patients with colorectal cancer before diagnosis. This increased granularity can help identify windows of opportunity for timely referral of patients presenting with alarm symptoms or prompt use of further triaging tests in patients with lower-risk symptoms.

### Implications for research and practice

An existing study suggests that diagnostic intervals >3 months may be associated with worse survival in some patients with cancer, including those with colorectal cancer.^26^ Other primary care studies examining pre-diagnostic activity in patients with urological cancer also used 3 months as a conservative cut-off to examine the timeliness of diagnosis.^27^^,^^28^ The implications of a time to diagnosis >3 months following an abnormal blood test are therefore considered here.

In this study, consultation rates for almost all examined clinical features (including gastrointestinal and constitutional symptoms, but excluding other bowel function symptoms) started increasing significantly >3 months pre-diagnosis in patients with colon cancer, suggesting that, in at least some patients, opportunities exist for an earlier initiation of cancer-specific investigations. For example, the rates of rectal bleeding and change in bowel habit increased from 10 and 8 months pre-diagnosis, respectively. Although it is possible that the increased diagnostic intervals were because of variations in the duration and intensity of symptom presentation that could not be fully captured by this study, it is likely that at least some patients with these two alarm symptoms would have qualified for a fast-track referral but did not receive one, resulting in the long diagnostic interval. This concurs with a recent study showing that GPs in England did not make timely expedited referrals for 82% of patients presenting with rectal bleeding.^29^ Other evidence also found that lack of knowledge of NICE referral criteria and concerns of over-referring contributed to the delayed referral of patients with abnormal colorectal cancer clinical features.^30^

The early increase in rates of consultation for abdominal pain at 8 months pre-diagnosis in patients with colon cancer may reflect the diagnostic challenges posed by the symptom’s low positive predictive value for cancer (that is, a less specific symptom for cancer).^31^ The findings in the current study suggest that some patients with colorectal cancer who present with less cancer-specific symptoms or non-alarm symptoms may benefit from further investigations available in primary care, such as FIT, as early as 8 months before diagnosis. There is therefore considerable opportunity to initiate cancer-specific investigations sooner to rule out cancer.

In patients with rectal cancer, consultation rates for change in bowel habit, rectal bleeding, and abdominal pain also increased significantly >3 months pre-diagnosis, suggesting that opportunities also exist for better triage of patients for further referral for definitive cancer investigations. Although patient- or system-level factors may also contribute to delays in diagnosis, it is unlikely that these factors will cause substantial delays once a referral (especially a fast-track referral) has been made.

Early increases in rates of all three examined blood tests were found in patients with both colon and rectal cancers. It is likely that further investigations using FIT could be useful in a significant proportion of patients who had low haemoglobin, which was reported as early as 9 months pre-diagnosis, to better identify those who would need further cancer investigations. Although high inflammatory markers and platelets are non-specific for cancer, the abnormality should prompt earlier investigative actions in at least a proportion of patients, especially in combination with other risk factors and abnormal clinical features or blood tests. It is worth noting that the predictive values of high inflammatory markers and platelets alone are not currently high enough to warrant a specialist referral under both the 2005 (in place at the time of the data collection in this study) and 2015 NICE guidelines.^14^^,^^32^^–^^34^ Therefore, a thorough systems enquiry and examination may be indicated when inflammatory markers and platelets are unexpectedly raised, and subsequent cancer-specific investigations performed if indicated. Further research into the impact of the lowering of referral threshold in the 2015 NICE guidelines on diagnostic intervals are in progress and will shed more light on the effect of these guidelines on cancer diagnosis.^35^

The findings of this study provide evidence for the existence of early signals of colorectal cancer-related symptoms and blood test abnormalities that should prompt appropriate further investigations or safety netting depending on the clinical context. Increasing GP awareness of the less cancer-specific symptoms and further characterisation of thresholds of abnormal blood tests (such as high platelets)^32^ may improve timely follow-up of symptoms and abnormal blood tests. The increased availability of FIT since the study period may also contribute to accelerations of cancer-specific investigations and referrals. Furthermore, the implementation of rapid diagnostic centres has the potential to expedite diagnosis of both cancer and non-cancer conditions in patients presenting with non-specific symptoms, and offer additional opportunities for reassurance and safety netting.^36^

In conclusion, this study found evidence for increasing rates of consultation for colorectal cancer-relevant symptoms and abnormal test results in the 2-year period before diagnosis. These findings showed that long diagnostic intervals of 8–9 months followed many colorectal cancer-relevant clinical features and abnormal blood tests. It is likely that a proportion of people who present with alarm symptoms such as rectal bleeding, change in bowel habit, and anaemia will benefit from more timely referrals for further investigations, and that windows of opportunity exist for earlier use of tests such as FIT for triaging patients for referral in those presenting with less specific symptoms and signs. This study demonstrated that there is scope to optimise timely referral for definitive diagnosis in patients with colorectal cancer who are symptomatic.

## Data

Data available from authors on request.
